# When Cronbach's alpha does (not) indicate the reliability of domain-specific knowledge tests and why

**DOI:** 10.3389/fpsyg.2026.1796702

**Published:** 2026-05-13

**Authors:** Steffen Zitzmann, Gabe A. Orona

**Affiliations:** 1 Department of Psychology, Medical School Hamburg, Hamburg, Germany; 2 Lynch School of Education and Human Development, Boston College, Chestnut Hill, MA, United States; 3 Hector Research Institute of Education Sciences and Psychology, University of Tübingen, Tübingen, Germany

**Keywords:** composite reliability, Cronbach's alpha, domain-specific knowledge test, McDonald's omega, measurement, reliability, test-retest reliability, variance inflation factor

## Introduction

It goes without saying, but we repeat it anyway, that the practice of measurement is crucial in the field of educational psychology. Also indisputable is that almost all concepts investigated and used in this field cannot be directly observed but can only be inferred from tests, questionnaires, and related instruments. Most of these devices were developed with the specific goal to be used in research. An important quality necessary for that purpose is that they are *accurate andreliable*—a point that we prominently made elsewhere (see Zitzmann et al., 2025, 2024a; Zitzmann, 2026). Authors of original research articles are generally encouraged to provide evidence on the reliability of their measures (American Educational Research Association et al., 2014), and domain-specific knowledge tests are no exception.

From the dominant perspective of reflective measurement, it is efficient to select items as indicators that are correlated with each other, as this strategy allows to maximize so-called internal consistency while minimizing the number of items needed to ensure a high level of reliability. However, scholars pointed out that such attempts can compromise validity through artificially narrowing down content (e.g., Loevinger, 1957; White, 2025). In the spirit of this critique, Edelsbrunner et al. (2025a) recently argued that valid domain-specific knowledge tests would typically exhibit low Cronbach's alphas (Cronbach, 1951; McNeish, 2018)—arguably the most widely used reliability coefficient in educational psychology—and that this indicates validity of these tests rather than unreliability. They concluded that alpha should no longer be interpreted as indicating the reliability of such tests.

Driven by concerns that this message could be easily exploited by others to justify why a low alpha was observed or not reported, Zitzmann and Orona (2025) commented on Edelsbrunner et al. (2025a) by presenting counterarguments against their view on the role of alpha in domain-specific knowledge tests. The counterarguments included that (a) there is no strong link between heterogeneity in content across items and a low alpha, thereby challenging conventional wisdom, (b) alpha assesses the reliability and not (only) the strength of interrelations among items, and (c) a low alpha can threaten the inferences from test scores and the trustworthiness of individual diagnostic judgements.

In response, Edelsbrunner et al. (2025b) defend their argument that alpha would not indicate the reliability of domain-specific knowledge tests. We are very grateful for their engagement, and thank them for their clarifications, which prompted us to rethink our argument. Essentially, they call our assumption that domain-specific knowledge tests tap into relatively unidimensional concepts into question, which they argue would be unwarranted due to the existing heterogeneity in content. To emphasize their point, the authors refer to Hofer et al. (2017), who developed a test of basic Mechanics Conceptual Understanding (bMCU) that Edelsbrunner et al. (2025b) believe would measure a very heterogeneous, if not even a formative concept.

In this rejoinder, we will elaborate on their reply, thereby further expanding our scholarly dialogue. Specifically, we will argue that content heterogeneity alone does not necessarily contradict unidimensionality or disqualify alpha. We will present three different readings of the term heterogeneity and their implications on dimensionality, arguing that only one reading challenges alpha as a reliability coefficient—the position that domain-specific knowledge is *formed* rather than reflected by items. At the same time, we are hesitant to believe that most domain-specific knowledge tests assess a formative concept. Therefore, we will critically examine the example presented by Edelsbrunner et al. (2025b) as support for their claim and ask whether the bMCU test really measures a formative concept.

## Content heterogeneity and its relation to unidimensionality and alpha

Edelsbrunner et al. (2025b) mainly criticize our commentary on their previous publication for assuming that domain-specific knowledge is relatively unidimensional, concluding our argument would not be warranted. (Edelsbrunner et al. 2025b) explain that “many studies show that ZaO's magenta Zitzmann and Orona, (2025) assumptions of [...] a relatively unidimensional construct are inappropriate for knowledge tests” (p. 2) because domain-specific knowledge is typically a “heterogeneous construct” (p. 3). From their argument, it is evident that Edelsbrunner et al. (2025b) assume that unidimensionality and content heterogeneity are inversely related, an assumption that calls for a critical examination. Indeed, many assessments (e.g., G-factor, Graduate Record Examinations, Law School Admission Test) entail some kind of content heterogeneity, but because heterogeneity does not equal unidimensionality, this per se does not preclude the use of alpha.

###  One knowledge domain with nuances in content

In our commentary on (Edelsbrunner et al. 2025a), taking the perspective of reflective measurement, we understood content heterogeneity as an inherent feature of a broad domain: items may vary in difficulty, with different levels of difficulty engaging different content within the domain. Similarly, items may differ in the extent to which cognitive processing is necessary. For example, some items may aim at reproducing knowledge (e.g., identifying a mechanical law), others may require test takers to apply this knowledge (e.g., solving a practical case by applying the law), and still others may be more evaluative (e.g., critically examining the law and qualifying its contexts of application). As a consequence, items measure unique aspects or nuances of domain-specific knowledge rather than being duplicates of each other (see Edelsbrunner et al., 2025a). This point was also made by Wilson in his book Constructing Measures and is evident in many examples as well. However, the best doctors, lawyers, or mechanics are those that do not just excel in one item but many items, leading to the assumptions that items are interrelated and likely correlated to some degree. As we noted, these correlations may not necessarily be strong, although this is often considered beneficial. Despite differing in content, the items can be considered interchangeable in the sense that they are sampled from the same knowledge domain, which provides the basis for interpreting the test score according to the meaning ascribed to this domain (e.g., as indicating a student's understanding of basic Newtonian mechanics). This defined our notion of unidimensionality, which is perfectly in line with classical test theory, domain sampling, and the generalizability theory perspective.

Whereas we assumed that items are mostly parallel, we acknowledged that this is not always a viable assumption, because a student's responses to the items might differ not mainly as a consequence of measurement error. We agreed that this can result in an alpha lower than the test's actual reliability (McNeish, 2018; Sijtsma, 2009; Cho, 2016; Revelle and Zinbarg, 2009). At the same time, there is simulated and empirical evidence that even when items were evidently non-parallel, alpha indicated the right level of reliability (e.g., Raykov and Marcoulides, 2023; Edwards et al., 2021; Savalei and Reise, 2019). An anonymous reviewer pointed us to another, previously overlooked limitation of alpha. They argued that items may not be designed to differentiate between test takers, but rather to determine how much that group knows about the domain. Consequently, the fact that the items were not intended to produce dispersion may lead to a low alpha (which does however not necessarily indicate low reliability).

###  Multiple factors, one standing out

In his seminal publication, Cronbach's (1951) refined the aforementioned classical view on content heterogeneity by employing factor analytic reasoning. Whereas he was very clear that “in a homogeneous test, the items measure the same things” (p. 320), he was less explicit about what will follow if the test consists of heterogeneous items. Nevertheless, he emphasized that measuring the same does not require that all items be “factorially similar,” it requires only that “a large proportion of the test variance be attributable to the principal factor running through the test” (p. 320), opening the possibility for multiple factors (see Sijtsma, 2009). This means that a test can be heterogeneous (i.e., multi-factorial) while at the same time being relatively unidimensional in the sense that differences in test scores are mostly due to one factor.

While alpha is not expected to lead to a serious understatement of reliability (Cronbach, 1951), other coefficients may be better suited. As Brunner and Süß (2005) noted, the choice of alternative depends on how the test score should be interpreted: as being a blend of different factors or as reflecting mainly one. For each mode of interpretation, there is a corresponding coefficient. The former mode calls for the composite reliability coefficient (Raykov and Shrout, 2002), while the latter corresponds to McDonald's (1999) omega.

Summing up so far, our and Cronbach's (1951) interpretations of content heterogeneity are both compatible with readings of unidimensionality that place the sampling from a single domain or the dominance of one factor into the center of their definition. In either case, alpha is a defensible, yet not always perfect choice to report reliability evidence (Zitzmann, 2026).

###  Not interchangeable content

However, there is another, third reading of content heterogeneity: rather than representing nuances or, alternatively, tapping into a concept with one dominant or superordinate factor, some have argued that distinct items would define or *form* rather than reflect domain-specific knowledge (see e.g., Stadler et al., 2021; White, 2025 for recent advocates of this view). A change in one item might impact knowledge but not the other way around: higher knowledge will not by definition result in higher scores on each item (Bollen and Lennox, 1991; Jarvis et al., 2003). Although Edelsbrunner et al. (2025b) do not use the word “formative,” it seems that they favor this interpretation.

We agree with them that if domain-specific knowledge was formative, alpha would indeed be difficult to defend and not an ideal solution. Rather, a different approach to reliability should be adopted, such as calculating the correlation between two subsequent test repetitions or checking for variance inflation to show that items contributed uniquely and made a substantial contribution to the test score.

However, a formative view is generally hard to defend when talking about psychological concepts. We still doubt that most domain-specific knowledge tests assess a formative concept. To emphasize this position, we will now discuss the example presented by Edelsbrunner et al. (2025b) and assess whether their argument for why the concept measured by the bMCU test is formative can really be considered valid.

## Why the bMCU test arguably measures a unidimensional reflective concept

The bMCU test is the result of a development process aiming at providing an efficient multiple choice test of understanding basic Newtonian mechanics. The test consists of 12 items representing different content areas, namely inertia and motion, force and acceleration, balance of forces, and reciprocal action.

###  Heterogeneous, yet unidimensional

In our commentary, we assumed that each item of a domain-specific knowledge test assesses a unique nuance. Items are still interchangeable, because they constitute a sample from the same knowledge domain. Edelsbrunner et al. (2025b) argue that this assumption would be unwarranted. Taking the bMCU test as an example, the authors point out that “the item texts are dissimilar and not interchangeable, as they tap into different facets of the construct” (p. 2).

Indeed, the bMCU items stem from four content areas, which suggests that the items can be grouped. Considering the authors emphasis on the difference between the item groups, it appears reasonable to assume that the bMCU test exhibits a strong multi-factorial structure. Empirically, the existence of such a structure should have translated into more than one eigenvalue of the correlation matrix that are significantly greater than 1, with the first eigenvalue being not much greater than the second one. However, the scree plot in Supplementary Figure S1 indicated that there was one dominating factor, which—among other evidence—led Hofer et al. (2017) to conclude that the bMCU test measures a unidimensional concept: “Consequently, how a student solved the items of the bMCU test depended solely upon the student's ability. Other systematic influences on the student's responses were ruled out and the assumption of one-dimensionality could be warranted” (p. 13). The bMCU test provides an excellent example for why content heterogeneity and varying item difficulties do not necessarily contradict unidimensionality.

###  Reflective, not necessarily formative

There are indications that Edelsbrunner et al. (2025b) believe the bMCU test would measure a formative concept. In this regard, it is interesting to note that the development of the bMCU test was guided by the basic understanding that “conceptual knowledge can be described as *abstract* and general” (Hofer et al., 2017, p. 2). This reminds us of the g in intelligence research, which too is abstract (as it does not refer to a concrete skill) and general (as it significantly influences most cognitive tasks)—both reasons why we typically consider g a reflective concept. Admittedly, the analogy does not provide any strong argument against Edelsbrunner et al. (2025b), but it casts first doubt that their view of the bMCU test is fully justified.

Edelsbrunner et al.'s (2025b) view becomes most obvious from their statement that “a student might have learned one facet but not another. Thus, it may be misleading to conclude that low item interrelatedness [...] indicates high measurement error (i.e., low reliability)” (p. 3). Indeed, interrelatedness is not necessary for formative concepts (Jarvis et al., 2003). Some scholars even pointed out that the absence of interrelations among items constitutes an advantage (e.g., Stadler et al., 2021).

However, we doubt whether this holds true for the concept underlying the bMCU test. Reasons for why this concept might rather be reflective came from Hofer et al. (2017) themselves. The authors stated that different content areas of basic Newtonian mechanics are assumed to be “*interrelated”* (Hofer et al., 2017, p. 2). This makes sense, because having understood basic Newtonian mechanics raises the likelihood to score high not only in one such area but all areas of the domain. While acknowledging that Hofer et al. (2017) were not very explicit about how these areas are interrelated, we interpret their statement as implying that items correlate across groups of items so that one area provides information for another. As interrelatedness is expected for reflective concepts (Jarvis et al., 2003), this fits better with the interpretation of the bMCU test as measuring a reflective concept.

Another reason that we think supports our view is Hofer et al.'s (2017) employed methodology, which is Item Response Theory (IRT). Scholars have long recognized that the Rasch model—the simplest within the class of item response theory models—and the prototypical approach for reflective modeling—factor analysis—both target correlations between items. For example, Lord and Novick (1968) already showed that viewing the binary items as resulting from discretizing unobserved continuous items with a one-factor structure, each such (binary) item is a *regression* on that factor, with the functional form being the normal ogive (see Takane and De Leeuw, 1987). That the item is regressed on the factor rather than vice versa is perfectly in line with the reflective interpretation according to which a student's response is caused by the underlying factor. In practice, fitting the Rasch model requires that items correlate. It does not work well with nearly uncorrelated items so that issues can be indicative of items measuring unrelated content, rendering a formative interpretation likely. Thus, when developers intend to validate if their test measures their unidimensional reflective concept, the Rasch model is one possibility.

Hofer et al. (2017) found that the Rasch model converged smoothly and that the bMCU items conformed to this model, giving no reason for doubting that the bMCU test measures a unidimensional reflective concept whereby alpha is relevant. It would have been interesting to learn about the alpha (or a better suited coefficient) of the bMCU test, but Hofer et al. (2017) did not report such classic reliability indices. We therefore calculated alpha ourselves based on the standardized loadings of the 12-item version (11-item version) of the bMCU test, as reported by Hofer et al. (2017) in Supplementary Table S1 (Supplementary Table S3, respectively). Consistent with our assumption that the bMCU test measures a reflective concept, we obtained an alpha value of 0.62 (0.61), which can be considered acceptable, especially for newly developed instruments in smaller studies (values of 0.7 or 0.8 are often required for established tests). Furthermore, this value is within the range of what can be expected based on studies using similar tests (Edelsbrunner et al., 2025a).

To sum up, while fully agreeing with Edelsbrunner et al. (2025b) and others that some knowledge tests measure a formative concept (e.g., when content is unrelated), we still doubt that most domain-specific knowledge tests assess a formative concept. The canonical formative concept is socioeconomic status, which is clearly formed by a composite of income, education, and related indicators that cannot be viewed as flowing from an inner quality of a person. To us, this seems like a hard sell for knowledge testing situations. Even the example presented by the authors does neither provide conclusive theoretical reason nor sufficient empirical evidence for a formative interpretation.

## Concluding remarks with practical recommendations

We have argued that internal consistency such as alpha is most commonly adopted in the field, and that alpha is a justifiable choice in most cases in which a domain-specific knowledge test targets a unidimensional reflective concept. These include tests comprised of items sampled from one broad domain with varying nuances in content, as well as tests exhibiting a weak multi-factorial structure with one factor standing out. However, when factor analysis indicates strong multidimensionality or considerably non-parallel items, alpha can be suboptimal. Due to known limitations of coefficient alpha in such situations, educational psychologists might complement their report on reliability evidence by better suited alternatives, such as composite reliability or omega, with a clear justification for their choice.

While questioning that most domain-specific knowledge tests measure a formative concept, we acknowledged that such tests exist. However, when authors refer to the formative nature of their concept, it is mandatory to present a strong and sound theoretical explanation why it is conceptualized as formative instead of reflective, preferably followed by an empirical argument. This requires them to think clearly about the concept underlying their measure, which is certainly a more demanding task than just referring to Edelsbrunner et al. (2025a) and similar sources. In addition, authors should still provide reliability evidence, such as test-retest reliability or variance inflation factors.

**Figure 1:**
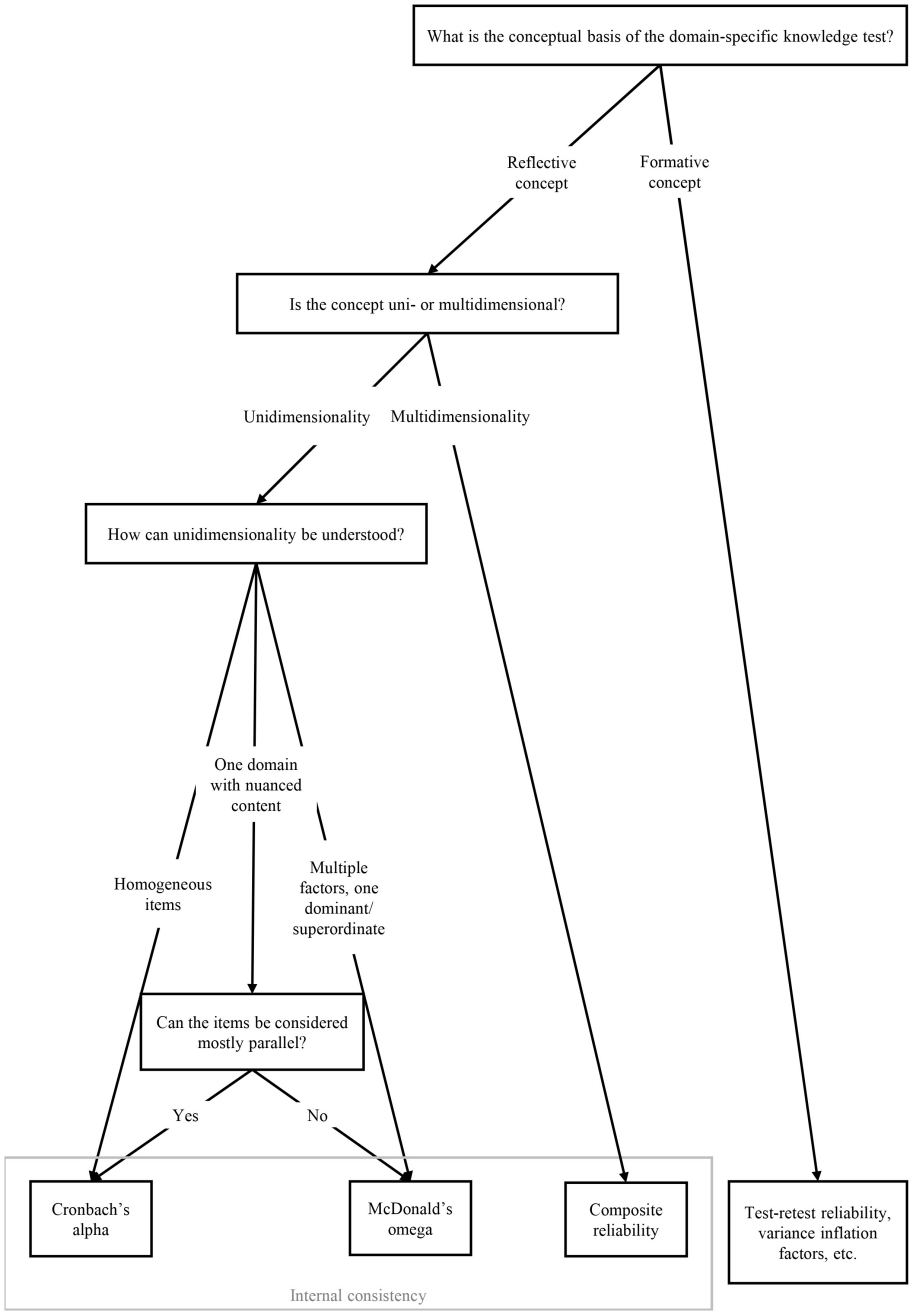
Decision tree to reliability evidence.

Figure 1 shows a decision tree designed to help readers translate our findings into concrete recommendations for reporting reliability evidence. However, we would like to emphasize that our guidelines should not be applied too mechanically. For example, alpha can serve as a lower bound for reliability even when items are non-parallel; and test-retest reliability can provide valuable additional reliability evidence beyond internal consistency.

Given the dominance of some scholars in the debate, one might be inclined to conclude that domain-specific knowledge tests do not capture reflective concepts, even though they are frequently analyzed as such. These authors have argued that domain-specific knowledge should theoretically be conceptualized as a formative concept. A close look at the example presented by Edelsbrunner et al. (2025b), the bMCU test, revealed that there is no evidence in favor of a formative interpretation, questioning the bMCU test's suitability as an example for formative measurement. We must admit that such counter-evidence can only be anecdotal and that we have not yet been able to definitively substantiate our actual argument that knowledge tests often capture reflective concepts. Therefore, we propose considering it rather as a research question or hypothesis, and thus as a starting point for an interesting follow-up study. Such a study could examine the theoretical foundations of published and unpublished knowledge tests to find arguments and empirical evidence for or against reflective conceptualizations. We are convinced that such evidence would support our hypothesis that the concepts underlying knowledge tests are more likely to be interpreted as reflective. Nevertheless, we would like to point out that as long as this evidence is lacking, our hypothesis is nothing more than an unconfirmed speculation, and we cannot rule out that it represents an overgeneralization and that our critics are right.

In conclusion, we wish to emphasize that there can be no “one size fits all” approach to reliability, and that we generally encourage a wide variety of different approaches (methodological pluralism; e.g., Zitzmann et al., 2024b; Zitzmann and Loreth, 2021). Acknowledging that one approach may be preferable to another, the choice of approach should be based on sound theoretical considerations and preferably supported by empirical evidence. We hope that researchers find our rejoinder on Edelsbrunner et al. (2025b) inspiring, motivating them to reflect on reliability more explicitly in the preparation of manuscripts covering issues in educational psychology and beyond.
